# Stability of Bioactive Compounds in Broccoli as Affected by Cutting Styles and Storage Time

**DOI:** 10.3390/molecules22040636

**Published:** 2017-04-15

**Authors:** Ana Mariel Torres-Contreras, Vimal Nair, Luis Cisneros-Zevallos, Daniel A. Jacobo-Velázquez

**Affiliations:** 1 Tecnológico de Monterrey, Escuela de Ingeniería y Ciencias, Centro de Biotecnología FEMSA, Av. Eugenio Garza Sada 2501 Sur, Monterrey 64849, Mexico; marieltorres2811@gmail.com; 2 Department of Horticultural Sciences, Texas A&M University, College Station, TX 77843-2133, USA; vimaln@tamu.edu (V.N.); lcisnero@tamu.edu (L.C.Z.)

**Keywords:** broccoli, phenolic compounds, glucosinolates, isothiocyanates, sulforaphane, ascorbic acid, cutting style, wounding stress

## Abstract

Broccoli contains bioactive molecules and thus its consumption is related with the prevention of chronic and degenerative diseases. The application of wounding stress to horticultural crops is a common practice, since it is the basis for the fresh-cut produce industry. In this study, the effect of four different cutting styles (CSs) (florets (CS1), florets cut into two even pieces (CS2), florets cut into four even pieces (CS3), and florets processed into chops (CS4)) and storage time (0 and 24 h at 20 °C) on the content of bioactive compounds in broccoli was evaluated. Immediately after cutting, 5-*O*-caffeoylquinic acid and caffeic acid content increased by 122.4% and 41.6% in CS4 and CS2, respectively. Likewise, after storage, 3-*O*-caffeoylquinic acid and 5-*O*-caffeoylquinic acid increased by 46.7% and 98.2%, respectively in CS1. Glucoerucin and gluconasturtiin content decreased by 62% and 50%, respectively in CS3; whereas after storage most glucosinolates increased in CS1. Total isothiocyanates, increased by 133% immediately in CS4, and after storage CS1 showed 65% higher levels of sulforaphane. Total ascorbic acid increased 35% after cutting in CS2, and remained stable after storage. Results presented herein would allow broccoli producers to select proper cutting styles that preserve or increase the content of bioactive molecules.

## 1. Introduction

Broccoli (*Brassica oleracea* L.) is a very important crop worldwide. Its production has increased ~400% from 1980 to 2015, in part due to consumer’s awareness of its health-promoting properties [1]. Broccoli’s health benefits are predominantly associated with its high content of bioactive compounds such as phenolics, glucosinolates, isothiocyanates, and ascorbic acid. Phenolics are widely recognized as potent antioxidant, cardioprotective, and anticarcinogenic compounds [2]. Glucosinolates are sulfur-rich amino acid derivatives considered as the most important secondary metabolite in broccoli due to one of their hydrolysis products (isothiocyanates), which possess potent anticarcinogenic properties related to their capacity to induce phase II enzymes, cell cycle arrest, and apoptosis [3,4,5,6,7]. From the different isothiocyanates, sulforaphane has been the most studied, and several nutraceutical properties such as the capacity to eradicate infections by *Helicobacter pylori* [8] and anti-inflammatory effects have been attributed to this molecule [9]. In addition, broccoli contains ascorbic acid, which is a nutritional compound that represents the first line of antioxidant defense in human plasma [10,11].

Today, the application of wounding stress to horticultural crops is a common practice, since it is the basis for the minimally processed or fresh-cut produce that appeared in the 1990s [12]. The application of wounding in crop plants results in the degradation or even the biosynthesis and accumulation of secondary metabolites with health-promoting properties. In this context, it has been well characterized that wounding stress induces the accumulation of phenolic compounds in plants through a complex cross-talk between signaling pathways [13]. Likewise, recently wounding was proposed as an effective strategy to increase the concentration of specific glucosinolates and phenolics in broccoli florets [14,15]. On the other hand, wounding and storage time have been reported to reduce the content of ascorbic acid in broccoli [16].

**Figure 1:**
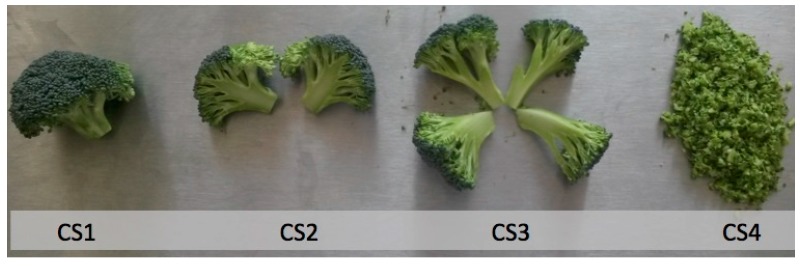
Different cutting styles (CSs) applied to broccoli heads: floret (CS1), floret cut into two even pieces (CS2), floret cut into four even pieces (CS3), florets processed into chops (CS4).

Little is known on the effect of wounding stress on the stability of bioactive compounds in broccoli. Therefore, the present project objective was to evaluate the effect of four different cutting styles (CSs) (Figure 1, florets (CS1), florets cut into two even pieces (CS2), florets cut into four even pieces (CS3), and florets processed into chops (CS4)) and storage time (0 and 24 h at 20 °C) on the concentration of phenolic compounds, glucosinolates, isothiocyanates, sulforaphane, and total ascorbic acid in broccoli. The information presented herein will help the fresh produce industry to select proper cutting styles that preserves the levels of bioactive compounds in broccoli. Likewise, this investigation will allow the elucidation of alternative methods to use broccoli as a biofactory of health-promoting properties when subjected to certain cutting styles.

## 2. Results and Discussion

### 2.1. Effect of Cutting Style and Storage Time on the Content of Phenolic Compounds

**Figure 2:**
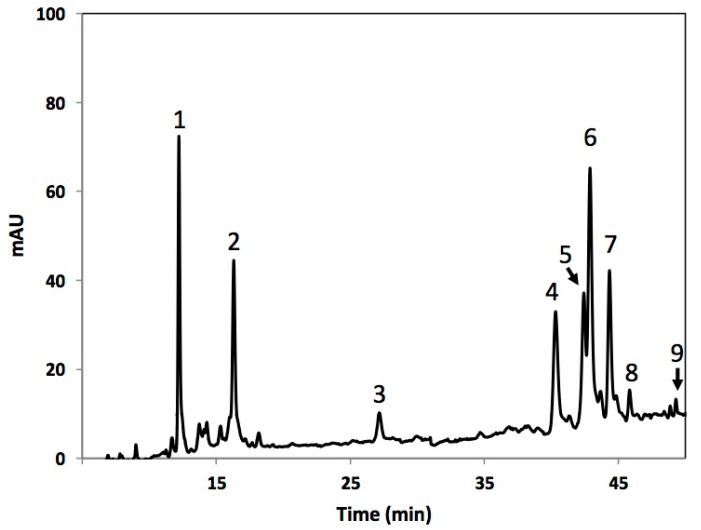
Typical high-performance liquid chromatography-diode array detection (HPLC–DAD) chromatogram (shown at 320 nm) of methanol extracts of broccoli. Tentative identification of peaks was performed as indicated in Table 1. Peak assignment: (1) 3-*O*-CQA; (2) 5-*O*-CQA; (3) CA; (4) 1-S-2-FG; (5) 1,2,2-TSG; (6) 1,2-DFG; (7) 1,2-DS-2-FG; (8) 1-2-2,2-DFG (9) 1,2,2-TSPG.

Individual phenolic compounds identified in broccoli heads subjected to four different cutting styles (Figure 1) are shown in Figure 2 and Table 1. Phenolic compounds identified included 3-*O*-caffeoylquinic acid (3-*O*-CQA), 5-*O*-caffeoylquinic acid (5-*O*-CQA), caffeic acid (CA), 1-sinapoyl-2-ferulolylgentiobiose (1-S-2-FG), 1,2,2-trisinapoylgentiobiose (1,2,2-TSG), 1,2-diferulolylgentiobiose (1,2-DFG), 1,2-disinapoyl-2-ferulolylgentiobiose (1,2-DS-2-FG), 1-sinapoyl-2,2-diferulolylgentiobiose (1-S-2,2-DFG), and 1,2,2-trisinapoylgentiobiose (1,2,2-TSPG). The phenolic profile identified in the present study is similar (qualitatively and quantitatively) to previous reports, where 3-*O*-CQA and 1,2-DFG were reported as the major phenolics in broccoli [15,17].

In general, the total phenolic content (calculated as the sum of the concentration of all individual phenolic compounds) was neither affected by the cutting style nor the storage time (Table 2). However, the interaction between the two variables affected the concentration. Immediately after cutting, total phenolic content was not affected in broccoli, whereas at 24 h of storage, phenolics increased by 53.2% in the florets and remained constant in the other CS when compared to time 0 h samples. For other crops, such as carrot and potato, higher increases (100%–300%) in total phenolics due to wounding stress have been reported [18,19,20]. It is well known that wounding stress induces oxidative stress in plant tissues [20]. Based on this, Reyes et al. [21] hypothesized that initial concentration of ascorbic acid dictates the degree of activation of the phenylpropanoid metabolism in fruits and vegetables, where phenolic compounds are synthesized. In this context, wounded crops with low levels of ascorbic acid (i.e., carrot and potato), rapidly consume ascorbic acid to neutralize free radicals (i.e., reactive oxygen species, ROS), and thus the biosynthesis of phenolics is highly activated to produce phenolic antioxidants needed to modulate ROS levels. Since broccoli contains higher levels of ascorbic acid compared with carrot and potato (see Section 2.4), the wound-induced accumulation of phenolics was lower when compared with values previously reported for these crops.

Regarding the individual phenolic compounds, the caffeoylquinic acid derivatives (3-*O*-CQA and 5-*O*-CQA) and CA were significantly affected by the cutting style and by the interaction between cutting style and storage time. In this context, the 3-*O*-CQA levels were not significantly affected immediately after cutting, whereas 5-*O*-CQA and CA content increased by 122.4% and 41.6% in CS4 and CS2, respectively (Table 2). After storage, the concentration of 3-*O*-CQA and 5-*O*-CQA increased by 46.7% and 98.2%, respectively in CS1. This result agrees with a previous report [15], where increases of 22% in 3-*O*-CQA were reported after storage (24 h at 20 °C) of broccoli florets.

On the other hand, the content of the sinapoyl derivatives 1-S-2-FG, 1,2-DS-2-FG, 1-S-2,2-DFG, and 1,2,2-TPG were not significantly affected immediately after cutting. However, after storage the levels of 1,2,2-TSG increased by 64.1% in CS3. Likewise, the content of 1,2-DFG increased by 60.19% and 67.9% in CS1 and CS3, respectively, after 24 h of storage, whereas for CS2 and CS4 the content remained unaltered.

The higher levels of 5-*O*-CQA and CA observed immediately after cutting in CS4 and CS2, respectively, could be related with changes in extractability due to cell wall disruption. It has been reported that particle size reduction lead to cell wall disruption, modifying the bioaccessibility of phenolic compounds in carrot [22], tomato [23], herbal teas [24], wheat bran [25,26], and other cereal grains [27]. Interestingly, a correlation was not observed between higher wounding intensity and higher extractability of 5-*O*-CQA and CA. It is important to point out that the resulting phenolic content observed in broccoli right after cutting must be the balance between increases in extractability and enzymatic oxidation. In this context, the degradation of 1,2-DFG observed in CS3 (14%, from 374.8 to 323.2 mg/kg dry weight (DW)) may be related to its enzymatic oxidation by polyphenol-oxidase (PPO), due to loss of cellular compartmentation between the phenolic compounds (mainly in the vacuole) and PPO (in the cytoplasm) [28].

Regarding the effects of storage time on the levels of individual phenolics in the different cutting styles, it was observed that CS1 showed the highest levels of certain phenolics (3-*O*-CQA, 5-*O*-CQA, 1-S-2-FG, 1,2,2-TSG, 1,2-DFG, 1,2-DS-2-FG), whereas the levels of other decreased (CA) or remained unaltered (1-S-2,2-DFG; 1,2,2-TPG). It is well known that the phenolic metabolism of plants is affected by wounding stress and storage time [12,13,15,18,19,20,21]. Phenolic metabolism is activated by wounding to produce phenolic compounds needed for the biosynthesis of lignin [29,30]. Therefore, the levels of phenolic compounds observed in wounded tissue are the result of a balance between its biosynthesis and utilization rate [18,19,21,30]. In this context, it is likely that the higher levels of total phenolics detected in CS1 could be related with a lower utilization rate of soluble phenolics for lignin biosynthesis as compared to CS2, CS3, and CS4, since highest wounding intensity would favor the lignification process [29].

### 2.2. Effect of Cutting Style and Storage Time on the Content of Glucosinolates

**Figure 3:**
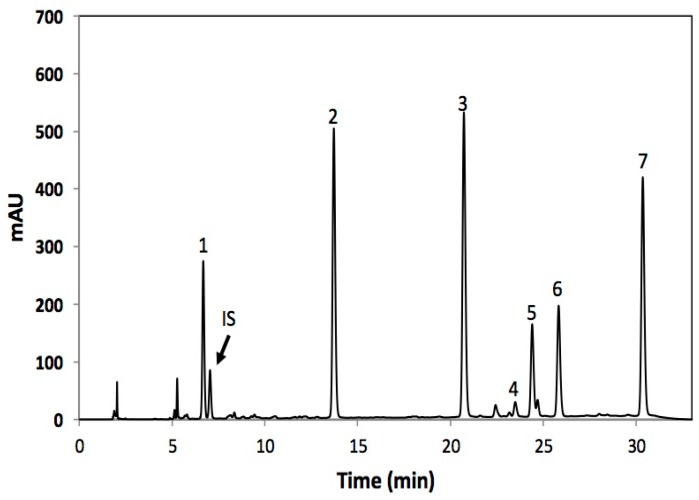
Typical HPLC–DAD chromatogram (shown at 227 nm) of desulfoglucosinolates obtained from methanol/water (70/30, *v*/*v*) extracts in broccoli. Tentative identification of peaks was performed as indicated in Table 3. Peak assignment: (1) Desulfoglucoraphanin; (2) 4-hydroxydesulfoglucobrassicin; (3) Desulfoglucoerucin; (4) Desulfoglucobrassicin; (5) Desulfogluconasturtiin; (6) 4-methoxydesulfoglucobrassicin; (7) Desulfoneoglucobrassicin; (IS) Internal standard (desulfosinigrin).

Glucosinolates identified in broccoli are shown in Figure 3 and Table 3. Individual glucosinolates identified included one aromatic (gluconasturtiin), two aliphatic (glucoraphanin and glucoerucin), and four indolic (4-hydroxy-glucobrassicin, glucobrassicin, 4-methoxy-glucobrassicin, and neo-glucobrassicin) glucosinolates. The glucosinolate profile obtained herein is similar to previous reports [14,15,17,31]. Previous studies have reported glucoraphanin [14,17] or glucobrassicin [15,31] as the major glucosinolate in broccoli. However, herein the most abundant glucosinolate was glucoerucin followed by neoglucobrassicin and glucoraphanin. Differences in the glucosinolate profiles reported herein and previous reports could be attributed to discrepancies between cultivars and growing conditions [31,32]. Glucoerucin has been reported as the second major glucosinolate present in the cultivar greendom [32] and in broccoli sprouts [33]. Furthermore, a broccoli cultivar line denominated “IL704.1” contains glucoerucin as its major glucosinolate [31].

The content of total glucosinolates, calculated as the sum of the concentration of all individual glucosinolates, was affected immediately after cutting in CS3, where about 50% of decrement was observed (Table 4). Regarding individual glucosinolates, the concentration of glucoerucin and gluconasturtiin decreased by 62% and 50%, respectively in CS3. This decrease may be related to transformation via enzymatic hydrolysis. Immediately after wounding, the plant cell is decompartmentalized allowing the interaction between glucosinolates and myrosinase (glucosinolate hydrolisis enzyme) causing their transformation [34].

In general, the content of all glucosinolates increased with storage time in CS1, showing the highest percentage of increase 4-hydroxy-glucobrassicin (1206%), followed by 4-methoxy-glucobrassicin (862%), glucoerucin (441%), gluconasturtiin (413%), neo-glucobrassicin (398%), and glucoraphanin (324%). Likewise, CS4 also showed increases in the content of certain glucosinolates such as the gluconasturtiin (602%), 4-hydroxy-glucobrassicin (429%), glucoerucin (218%), neoglucobrassicin (212%), and glucoraphanin (92%). These results agree with previous reports where wounding induce an increase in the concentration of indole glucosinolates [14,15,35]. The wound-induced accumulation of glucosinolates observed in broccoli may be associated with the upregulation of *BoCYP83B1* gene, which is involved in the biosynthesis of indolic and aromatic glucosinolate. This gene has been reported to be induced by signal transduction pathways triggered by wounding, where jasmonic acid function as a signaling molecules [35,36]. Furthermore, wounding also induce transcription factors (MYB51, MYB122, and MYB34) related to indolic glucosinolate biosynthesis [37]. Likewise, the larger accumulation of indole glucosinolates observed during storage as compared to aliphatic and aromatic glucosinolates may be related with the wound-induced activation of peroxidases [38], which catalyzes the rate limiting step in the conversion of tryptophan to indole glucosinolates [39].

### 2.3. Effect of Cutting Style and Storage Time on the Content of Total Isothiocyanates and Sulforaphane

**Figure 4:**
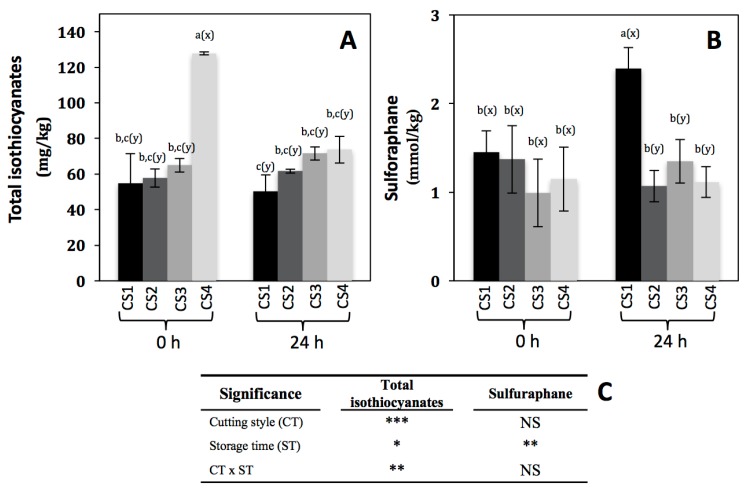
Concentration of total isothiocyanates (**A**) and sulforaphane (**B**) in broccoli subjected to different cutting styles and stored for 0 and 24 h at 20 °C. Cutting styles (Figure 1): floret (CS1), floret cut into two even pieces (CS2), floret cut into four even pieces (CS3), florets processed into chops (CS4). (**C**) Full factorial analyses of variance showing the main effects and interactions of the variables evaluated. Data represents the mean of three repetitions ± standard error of the mean. Different letters among bars indicate statistical difference by the LSD test (*p* < 0.05). (a–c) when comparing all treatments and storage times, (x-y) when comparing all treatments at the same storage time (0 h or 24 h). Asterisks indicate that main effects and interactions are significantly different by analyses of variance (ANOVA). NS—non significant, * *p* < 0.05, ** *p* < 0.01, *** *p* < 0.001.

Isothiocyanates are one of the hydrolysis products of glucosinolates after the action of myrosinase (thioglucoside glucohydrolase, EC 3.2.1.1), and are involved in the prevention of different chronic and degenerative diseases [40]. The total isothiocyanates content is shown in Figure 4A. As expected, immediately after cutting, total isothiocyanates increased by 133% (from 54.77 to 127.8 mg/kg) in CS4 as compared to CS1. This must be due to the action of myrosinase, since after wounding the plant cell is decompartmentalized allowing the interaction between glucosinolates and myrosinase [34]. In addition to total isothiocyanates, the content of a specific isothiocyanate (sulforaphane) was evaluated (Figure 4B), because this compound has been highly associated with the prevention of cancer [3,4,5,6,7], and is a hydrolysis product of glucoraphanin, one of the major glucosinolates in broccoli. Interestingly, sulforaphane content remained unaltered immediately after the application of the different cutting styles in broccoli (Figure 4B), whereas the content of total isothiocyanates increased in CS4 (Figure 4A). This can be explained in terms of the kind of glucosinolate being hydrolyzed immediately after cutting. As observed in Table 4, the content of glucoraphanin remained unaltered after cutting, whereas glucoerucin decreased 62% in CS3 as compared with CS1, suggesting that other isothiocyanates such as erucin (the hydrolysis product of glucoerucin) instead of sulforaphane are being produced immediately after cutting.

Regarding the effect of storage time on the total isothiocyanate content in broccoli subjected to different cutting styles (Figure 4A), the levels remained unaltered in CS1, CS2, and CS3, however, for CS4 the content decreased by 42% (from 127.8 to 73.67 mg/kg). On the other hand, sulforaphane showed 65% of increase (from 1.45 to 2.39 mmol/kg) in CS1, whereas the content did not change in the other cutting styles after storage (Figure 4B). This can be explained in terms of isothiocyanate stability to different factors such as oxygen [41]. In the case of total isothiocyanates, the compounds produced after cutting in CS4 were susceptible to storage conditions, since this cutting style is highly exposed to oxygen due to the higher reduced particle size as compared to the other CSs. Interestingly, in the case of sulforaphane its glucosinolate precursor (glucoraphanin) also increased in CS1 after storage (Table 4). Thus, results indicate that the biosynthesis rate of glucoraphanin is higher than its conversion to sulforaphane. Likewise, the accumulation of sulforaphane detected in CS1 is due to higher production rate than its degradation rate as compared with the other cutting styles.

### 2.4. Effect of Cutting Style and Storage Time on Ascorbic Acid Content

**Figure 5:**
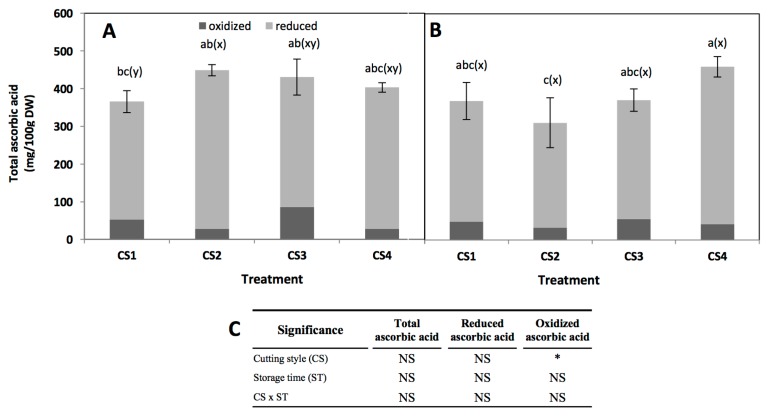
Total, reduced (AA), and oxidized (DHAA) ascorbic acid concentration in broccoli subjected to different cutting styles (**A**) and stored for 24 h at 20 °C (**B**). Cutting styles (Figure 1): Floret (CS1), floret cut into two even pieces (CS2), floret into four even pieces (CS3), florets processed into chops (CS4). (**C**) Full factorial analyses of variance showing the main effects and interactions of the variables evaluated. Data represents the mean of three repetitions ± standard error of the mean. Different letters among bars indicate statistical difference by the LSD test (*p* < 0.05). (a–c) when comparing all treatments and storage times, (x-y) when comparing all treatments at the same storage time (0 h or 24 h). Asterisks indicate that main effects and interactions are significantly different by analyses of variance (ANOVA). NS—non significant, * *p* < 0.05, ** *p* < 0.01, *** *p* < 0.001.

Total, reduced (ascorbic acid, AA), and oxidized (dehydroascorbic acid, DHAA) ascorbic acid content is shown in Figure 5. Since DHAA can be easily converted into AA in the human body it is important to measure both AA and DHAA when evaluating ascorbic acid activity [10]. Cutting style, storage time, and their interaction did not show a significant effect on the total ascorbate content of broccoli (Figure 5). The ascorbic acid concentration obtained herein (~400 mg/100 g DW) is similar to values previously reported for broccoli, considering the variation among cultivars (between 57.35 to 131.35 mg/100 g fresh weight) [42,43]. Likewise, results obtained herein agree with a previous report indicating that the ratio of DHAA/AA varies between 5% to 26% during storage of broccoli at 20 °C for four days [42].

A slight increment (35%, from 312.6 to 421.4 mg/100 g DW) was observed in total ascorbic acid immediately after cutting in CS2, but no changes were observed in CS3 and CS4 (Figure 5A). This increment may be related with an increase in extractability due to cell disruption. In the CS3 and CS4 this phenomenon was not observed, indicating that ascorbic acid is rapidly used to maintain redox status in the tissue. After storage, only CS2 showed a decrease in total ascorbic acid content, while in the other cutting styles the levels remained unaltered. Changes in the concentration of ascorbic acid during storage would be related to the recycling process, since it has been reported that some genes involved in ascorbic acid recycling are induced by mechanical wounding [44,45].

## 3. Materials and Methods

### 3.1. Chemicals

Sulfatase from *Helix pomatia*, sinigrin hydrate, l-ascorbic acid (AA), trichloroacetic acid (TCA), dl-dithiothreitol (DTT), N-ethylmaleimide (NEM), 2,2′-bipyridyl, iron (III) chloride (FeCl_3_), 3-*O*-CQA (3-*O*-caffeoylquinic acid), 5-*O*-CQA (5-*O*-caffeoylquinic acid), butyl-isothiocyanate, caffeic acid (CA), 1,2-benzenedithiol, ß-mercaptoethanol (HPLC grade), methylene chloride (HPLC grade), sephadex A-25, sephadex C-25, acetonitrile (HPLC grade), methanol (HPLC grade), sulforaphane, sodium acetate, potassium phosphate, and orthophosphoric acid (H_3_PO_4_) were obtained from Sigma Chemical Co. (St. Louis, MO, USA). Desulfoglucoraphanin was obtained from Santa Cruz Biotechnology (Dallas, TX, USA).

### 3.2. Plant Material, Processing, and Storage Studies

Broccoli (*Brassica oleracea* L. var. Heritage) was harvested in Aguascalientes, México, in May, 2016, and obtained in Monterrey, N.L. México by a local distributor. The samples were washed and disinfected with chlorinated water (200 ppm, pH 6.5). Broccoli heads were subjected to the following cutting styles (CS): florets (CS1), floret cut into two even pieces (CS2), florets cut into four even pieces (CS3), and florets processed into chops (CS4) as shown in Figure 1. Three broccoli heads were used to prepare each cutting style. CS1, CS2, and CS3 were obtained using a commercial straight-edged knife, whereas CS4 was obtained with a food processor (Waring Commercial, WFP11, Torrington, CT, USA). Samples were stored inside hermetic plastic containers with periodic ventilation to avoid CO_2_ accumulation over 0.5% (*v*/*v*). Three biological replicates were performed for each treatment. All samples were stored for 24 h in an incubator (VWR, Radnor, PA, USA) at 20 °C, and the concentration of individual phenolics, individual glucosinolates, total isothiocyanates, sulforaphane, and ascorbic acid was determined before and after storage. Collected samples were freeze-dried (Labconco, Kansas City, MO, USA) to obtain broccoli powder prior to their analysis.

### 3.3. Analysis of Phenolic Compounds by High-Performance Liquid Chromatography–Diode Array Detection and High-Performance Liquid Chromatography–Electrospray Ionization Mass Spectrometry

For the extraction of phenolic compounds 200 mg of broccoli powder was homogenized with methanol (5 mL) using a tissuemizer (Advanced homogenizing system, VWR, Radnor, PA, USA) and then centrifuged (10,000× *g*, 1 h, at 4 °C). The clear supernatant (methanol extract) was filtered using nylon membranes (0.45 μm, VWR) before injection to chromatographic system. Individual phenolic compounds were identified and quantified by HPLC–DAD and HPLC–ESI–MS^n^ according to the method described by Villarreal-García et al. [15]. The identification of individual phenolic compounds was based on their retention time, UV spectra, and mass-to-charge ratio as compared with authentic standards and previous reports [15,17]. For the quantification of phenolic compounds, a standard curve of 5-*O*-CQA was prepared in the range of 0.5–100 ppm. The concentration of phenolics was expressed as mg of 5-*O*-CQA equivalents per kg of broccoli dry weight (DW).

### 3.4. Analysis of Glucosinolates by High-Performance Liquid Chromatography–Diode Array Detection and High-Performance Liquid Chromatography–Electrospray Ionization Mass Spectrometry

The glucosinolate extraction, desulfation, and analysis was performed as previously described [15,46]. Briefly, 10 mL of methanol:water (70:30, *v*/*v*), previously heated for 10 min at 70 °C, were added to 0.2 g of broccoli powder followed by 50 μL of a 3 mM solution of sinigrin as internal standard. Samples were vortexed and incubated at 70 °C for 30 min to ensure myrosinase inactivation. Afterward, extracts were left to cool at room temperature and centrifuged (3000× *g*, 5 min, 4 °C). Glucosinolates were desulfated and purified using disposable polypropylene columns (Thermo Fisher Scientific, Waltham, MA, USA) as previously described [15,46]. Desulfoglucosinolates were analyzed by HPLC–DAD and HPLC–ESI–MS^n^.

The identification and quantification of desulfoglucosinolates was performed with the chromatographic method previously described by Villarreal-García et al. [15]. Individual desulfoglucosinolates were identified based on retention time, ultraviolet (UV) spectra, and their mass-to-charge ratio as compared with authentic standards and previous reports [15,17]. Individual glucosinolate concentrations were calculated using the response factor methodology to correct for absorbance differences between desulfosinigrin and the other desulfoglucosinolates [47].

### 3.5. Analysis of Total Isothiocyanates by Ciclocondensation with 1,2-bezenedithiol

The extraction and analysis of total isothiocyanates was performed as described by Zhang et al. [48] with some modifications. Broccoli powder (75 mg) was suspended in 1.5 mL of water, mixed, and centrifuged at 12,000× *g* for 5 min (Centrifuge 5417R, Eppendorf, Hamburg, Germany) in the absence of light. Supernatants were decanted into a 2 mL microcentrifuge tube and used as extracts for cyclocondensation reactions.

The cyclocondensation reactions were carried out in 2 mL amber HPLC autosampler vials (1260 Infinity, Agilent Technologies, Santa Clara, CA, USA). The reaction mixture consisted of 0.8 mL of 100 mM potassium phosphate at pH 8.5, 0.4 mL of methanol containing 1,2-benzenedithiol (4.0 mM final concentration), and 0.4 mL of extract to be analyzed. Solutions were microfiltered using nylon membranes (0.45 μm) (VWR) before the reaction. The vials were flushed with nitrogen, and incubated for 2 h at 65 °C (Vortemp 1550, Labnet International, Woodbright, NJ, USA). Then vials were left to cool at room temperature. Separation was done on a 4.6 × 250 mm, 5 μm particle size, C18 reverse phase column (Luna, Phenomenex, Torrance, CA, USA).

The column was operated isocratically with methanol:water (80:20, *v*/*v*), at a rate of 1.5 mL/min for 15 min. The eluates were monitored at 365 nm by the DAD, and the area of 1,3-benzodithiole-2-thione peak (cyclocondensation product of 1,2-benzenedithiol with isothiocyanate) was integrated. A standard curve of 1,3-benzodithiole-2-thione derived from sulfuraphane in the range of 0–50 ppm was prepared to quantify total isothiocyanates. Results were reported as mg of total isothiocyanates per kg of broccoli DW.

### 3.6. Analysis of Sulforaphane

The extraction and analysis of sulforaphane was carried out according to the method reported by Ku et al. [49] with some modifications. Broccoli powder (75 mg) was suspended in 1.5 mL of water, mixed vigorously and centrifuged at 12,000× *g* for 5 min in the absence of light. The supernatant was recovered in a 2 mL tube and used as extract. Thereafter, 0.5 mL of extract was mixed with 0.5 mL of derivatization reagent (20 mM triethylamine with 200 mM ß-mercaptoethanol in methylene chloride) and 5 μL of butyl isothiocyanate (0.5 mg/mL) as an internal standard. Derivatization reaction was first incubated at room temperature during 24 h in a small tube rotator (Glas-Col, Terre Haute, IN, USA) operating at 50 percent of speed, and then it was incubated at 30 °C for 60 min under constant stirring. Finally, it was dried under a stream of nitrogen. The residue was dissolved in 200 μL of acetonitrile:water (1:1) (*v*/*v*) and 10 μL of the extract were injected in the HPLC-DAD system. Mobile phase consisted in water (phase A) and methanol (phase B). The gradient solvent system was 0/100, 10/90, 35/0, 40/0, and 50/100 (min/% phase A). Mobile phase B was 0% at injection, increasing to 10% by 10 min, 100% at 35 min, and held 5 min, then decreased to 0% by 50 min. Flow rate of the mobile phase was kept at 0.8 mL/min. The detector wavelength was set at 227 and 271 nm. A standard curve of sulforaphane was prepared in the range of 0–100 ppm for quantification, and results were expressed as mmol per kg of broccoli DW.

### 3.7. Extraction and Quantification of Reduced, Oxidized, and Total Ascorbate

Reduced, oxidized, and total ascorbic acid were determined using the 2,2′-bipyridyl method [50]. Briefly, dried broccoli (0.1 g) was homogenized with TCA (6%, 5 mL), transferred to a 15 mL tube, centrifuged (15,000× *g*, 50 min, 4 °C) and maintained on ice until needed. For total AA, the extract (100 μL) was placed in a 2-mL tube and mixed with DTT solution (20 mM, 100 μL). The mixture was incubated for 10 min at room temperature in absence of light. Then, NEM solution (0.5%, 100 μL) was added to the mixture and incubated for 30 s. For reduced AA, the extract (100 μL) was placed in 2-mL tube and mixed with water (200 μL) instead of DTT and NEM solutions. Subsequently, TCA (10%, 500 μL), H_3_PO_4_ (43%, 400 μL), 2,2′-bipyridyl (4%, 400 μL), and FeCl_3_ (3%, 200 μL) solutions were added to the total and reduced AA assay tubes. The assay tubes were incubated at 37 °C for 1 h. Then, 200 μL of the reaction solutions from the assay tubes were placed in a well of a clear 96-well microplate and absorbance readings were collected at 525 nm. Absorbance values were compared against an AA standard curve (0.15–10 mM) prepared in TCA (6%). Oxidized ascorbic acid was calculated as the difference between the total and reduced AA. Results were expressed as mg of reduced (AA), oxidized (DHAA), and total ascorbic acid per 100 g of broccoli DW.

### 3.8. Statistical Analysis

Replicates were achieved by repeating treatment under the same conditions. All treatments were run concurrently. All reported data were from three replicates per treatment (*n* = 3). Statistical analyses were performed using the three replicates. Data represent the mean values of samples and their standard error. Analyses of variance (ANOVA) were conducted to determine the main effects and interactions using JMP software version 9.0 (SAS Institute Inc., Cary, NC, USA) and mean separations performed using the LSD test (*p* < 0.05).

## 4. Conclusions

In the present study, it was shown that cutting styles affect the stability and accumulation of bioactive compounds in broccoli. The compounds that were affected the most by the application of wounding in broccoli were the glucosinolates, where significant increases in their concentrations were obtained in the florets stored for 24 h at 20 °C. Likewise, the florets showed increases in the content of phenolic compounds after storage. Interestingly, immediately after cutting broccoli, the concentration of certain compounds increased, suggesting that wounding increases the extractability and thus the bioavailability of bioactive compounds. The information presented herein will help the fresh produce industry and consumers to select proper cutting styles that preserve and increase the levels of bioactive compounds in broccoli. According with the results obtained herein, CS4 would be the optimum cutting style if broccoli is going to be consumed immediately after cutting, since the extractability of total isothiocyanates and phenolic compounds would be increased. On the other hand, if higher levels of nutraceuticals are desirable in the crop, broccoli florets (CS1) should be stored for 24 h at 20 °C to increase its total phenolic, glucosinolate and sulforaphane content before consumption. Further research should be focused on evaluating postharvest technologies that allow the commercialization of fresh-cut broccoli under proper CS, considering the high respiration rates, ethylene emissions, and related quality losses of the product during storage. Likewise, this investigation will allow the elucidation of alternative methods to use broccoli as a biofactory of health promoting properties when subjected to certain cutting styles.

## Figures and Tables

**Table 1:** Tentative identification of individual phenolic compounds in broccoli.

Peak Number (Retention Time in min)	λ Max (nm)	Tentative Identification	[M − Z]^−^ (*m*/*z*)	MS Fragments ^c^
1 (12.8)	295, 320	3-*O*-CQA ^a,b^	353	**179**, 173, 135
2 (16.4)	295, 320	5-*O*-CQA ^a,b^	353	**172.5**, 110.6, 92.7, 84.8
3 (27.5)	225, 325	CA ^b^	179	**179**, 161, 143, 133, 105
4 (40.6)	235, 320	1-S-2-FG ^b^	723	**449**, **223**
5 (42.7)	215, 235, 320	1,2,2-TSG ^b^	959	735, **223**
6 (43.1)	235, 320	1,2-DFG ^b^	693	499, **175**
7 (44.6)	215, 235, 320	1,2-DS-2-FG ^b^	929	705, **223**
8 (46.0)	215, 235, 320	1-S-2,2-DFG ^b^	899	705, **223**
9 (49.4)	235, 320	1,2,2-TSPG ^b^	959	735, **223**

Identification was obtained by high-performance liquid chromatography–electrospray ionization- mass spectrometry (HPLC–DAD–ESI–MS^n^). ^a^ Identified based on their spectra characteristics and the mass-to-charge ratio as compared with authentic standards. ^b^ Identified based on their spectra characteristics and their mass-to-charge ratio as compared with previous reports [15,17]. ^c^ Major fragment ions are shown in bold. 3-*O*-caffeoylquinic acid (3-*O*-CQA); 5-*O*-caffeoylquinic acid (5-*O*-CQA); caffeic acid (CA); 1-sinapoyl-2-ferulolylgentiobiose (1-S-2-FG); 1,2,2-trisinapoylgentiobiose (1,2,2-TSG); 1,2-diferulolylgentiobiose (1,2-DFG); 1,2-disinapoyl-2-ferulolylgentiobiose (1,2-DS-2-FG); 1-sinapoyl-2,2-diferulolylgentiobiose (1-S-2,2-DFG); and 1,2,2-trisinapoylgentiobiose (1,2,2-TSPG).

**Table 2:** Concentration of phenolic compounds in broccoli subjected to different cutting styles before and after 24 h of storage at 20 °C.

**Storage Time**	**Cutting Style ^A^**	**Phenolic Concentration (mg/kg DW) ^B,C,D,E^**									
		**3-*O*-CQA**		**5-*O*-CQA**		**CA**		**1-S-2-FG**		**1,2,2-TSG**	
0 h	CS1	321.7 ± 17.42	bc (x)	180.5 ± 56.7	bc (y)	267.4 ± 14.4	b (y)	233.3 ± 16.9	bc (x)	250.8 ± 16.1	abc (x)
	CS2	414.8 ± 51.2	ab (x)	240.7 ± 15.9	b (y)	378.9 ± 12.8	a (x)	282.2 ± 46.8	abc (x)	294.4 ± 45.0	abc (x)
	CS3	431.8 ± 58.7	ab (x)	171.9 ± 9.9	bc (y)	252.4 ± 63.8	bc (y)	289.0 ± 71.0	abc (x)	219.5 ± 34.7	bc (x)
	CS4	353.8 ± 12.9	b (x)	401.5 ± 21.3	a (x)	161.3 ± 7.8	cd (y)	277.0 ± 5.6	abc (x)	267.0 ± 26.6	abc (x)
24 h	CS1	472.0 ± 49.4	a (x)	357.8 ± 31.3	a (x)	114.1 ± 19.3	d (z)	369.9 ± 40.3	a (x)	359.4 ± 62.9	a (x)
	CS2	424.5 ± 42.5	ab (x)	143.4 ± 33.0	c (y)	267.6 ± 31.0	b (y)	308.5 ± 40.9	ab (x)	345.8 ± 57.3	ab (xy)
	CS3	488.6 ± 0.5	a (x)	183.6 ± 30.6	bc (y)	368.9 ± 39.3	a (x)	304.4 ± 6.6	ab (x)	360.3 ± 6.8	a (x)
	CS4	237.4 ± 11.8	c (y)	350.2 ± 15.2	a (x)	138.2 ± 6.6	d (z)	192.7 ± 6.0	c (y)	210.2 ± 9.0	c (y)
**Significance**											
Cutting style (CS)		**		***		***		NS		NS	
Storage time (ST)		NS		NS		NS		NS		**	
CS × ST		*		**		**		NS		NS	
**Storage Time**	**Cutting Style ^A^**	**Phenolic Concentration (mg/kg) ^B,C,D,E^**									
		**1,2-DFG**		**1,2-DS-2-FG**		**1-S-2,2-DFG**		**1,2,2-TPG**		**Total Phenolics**	
0 h	CS1	374.8 ± 13.8	cd (x)	235.2 ± 32.5	bc (x)	61.8 ± 4.3	ab (x)	35.1 ± 0.5	c (x)	1668.9 ± 116.9	bc (x)
	CS2	443.8 ± 39.4	bc (x)	259.4 ± 39.8	abc (x)	64.7 ± 7.5	ab (x)	35.9 ± 1.9	bc (x)	2213.5 ± 272.4	ab (x)
	CS3	323.2 ± 9.7	d (y)	330.5 ± 77.7	ab (x)	78.7 ± 16.5	ab (x)	46.2 ± 6.9	abc (x)	2140.7 ± 376.8	abc (x)
	CS4	398.3 ± 5.4	cd (x)	232.8 ± 22.7	bc (x)	63.5 ± 3.3	ab (x)	36.7 ± 3.2	bc (x)	1987.9 ± 69.6	abc (x)
24 h	CS1	600.4 ± 60.9	a (x)	367.4 ± 57.4	ab (x)	79.8 ± 11.4	ab (xy)	45.8 ± 4.2	abc (x)	2558.1 ± 272.4	a (x)
	CS2	520.5 ± 59.9	ab (x)	379.1 ± 64.0	a (x)	76.7 ± 11.8	ab (xy)	47.6 ± 4.6	ab (x)	2312.6 ± 247.2	a (x)
	CS3	542.7 ± 2.5	ab (x)	349.4 ± 33.3	ab (x)	83.4 ± 3.2	a (x)	49.3 ± 5.9	a (x)	2528.6 ± 64.2	a (x)
	CS4	322.6 ± 13.8	d (y)	177.9 ± 6.90	c (y)	53.7 ± 1.8	b (y)	35.8 ± 0.4	bc (x)	1513.4 ± 64.1	c (y)
**Significance**											
Cutting style (CS)		**		NS		NS		**		NS	
Storage time (ST)		**		NS		NS		NS		NS	
CS × ST		**		NS		NS		NS		*	

^A^ Cutting styles (Figure 1): floret (CS1), floret cut into two even pieces (CS2), floret into four even pieces (CS3), florets processed into chops (CS4). ^B^ Concentrations are reported as 5-*O*-CQA equivalents. All compounds were quantified at 320 nm. ^C^ Data represents the mean of three repetitions ± standard error of the mean. ^D^ Different letters in the same column indicate statistical difference by the least significant difference (LSD ) test (*p* < 0.05). (a-d) when comparing all treatments and storage times, (x-z) when comparing all treatments at the same storage time (0 h or 24 h). ^E^ Asterisks indicate that main effects and interactions are significantly different by analyses of variance (ANOVA). NS—non significant, * *p* < 0.05, ** *p* < 0.01, *** *p* < 0.001. Abbreviations: cutting style (CS); dry weight (DW); 3-*O*-caffeoylquinic acid (3-*O*-CQA); 5-*O*-caffeoylquinic acid (5-*O*-CQA); caffeic acid (CA); 1-sinapoyl-2-ferulolylgentiobiose (1-S-2-FG); 1,2,2-trisinapoylgentiobiose (1,2,2-TSG); 1,2-diferulolylgentiobiose (1,2-DFG); 1,2-disinapoyl-2-ferulolylgentiobiose (1,2-DS-2-FG); 1-sinapoyl-2,2-diferulolylgentiobiose (1-S-2,2-DFG); 1,2,2-trisinapoylgentiobiose (1,2,2-TSPG).

**Table 3:** Tentative identification of individual desulfoglucosinolates in broccoli.

Peak Number (Retention Time, min)	λ Max (nm)	Tentative Identification	[M − Z]^−^ (*m*/*z*)	MS Fragments ^d^
1 (6.6)	222	Desulfoglucoraphanin ^a,b,c^	356	**193**
2 (13.0)	222	4-hydroxydesulfoglucobrassicin ^b,c^	383	**221**, 203, 153
3 (20.8)	222, 280	Desulfoglucoerucin ^b,c^	340	**177**, 160, 129, 113
4 (23.5)	222, 280	Desulfoglucobrassicin ^b,c^	367	**204**, 187, 155, 129
5 (24.3)	222, 265, 280	Desulfogluconasturtiin ^b,c^	342	**179**, 162, 130, 104
6 (25.8)	222, 290	4-methoxydesulfoglucobrassicin ^b,c^	397	**234**, 204, 154, 139
7 (30.3)	222, 290	Desulfoneoglucobrassicin	397	**234**, 204, 154, 129

Identification was obtained by HPLC–DAD–ESI–MS^n^. ^a^ Identified based on their spectra characteristics and their mass-to-charge ratio as compared with authentic standards. ^b^ Identified based on their spectra characteristics and order of elution as compared with a previous report [15,17]. ^c^ Identified based on their spectra characteristics and their mass-to-charge ratio as compared with a previous report [17]. ^d^ Major fragment ions are shown in bold.

**Table 4:** Concentration of glucosinolates in broccoli subjected to different cutting styles before and after 24 h of storage at 20 °C.

**Storage Time**	**Cutting Style ^A^**	**Glucosinolate Concentration (μmol/kg DW) ^B,C,D,E^**							
		**Glucoraphanin**		**4-Hydroxy Glucobrassicin**		**Glucoerucin**		**Glucobrassicin**	
0 h	CS1	1081 ± 87	bc (w)	210 ± 21	d (wx)	2098 ± 240	bc (w)	116 ± 13	a (x)
	CS2	782 ± 162	cd (wx)	173 ± 43	d (wx)	1771 ± 434	cd (wx)	95 ± 12	a (x)
	CS3	617 ± 13	d (x)	255 ± 11	d (w)	793 ± 53	d (y)	121 ± 7	a (x)
	CS4	618 ± 43	d (x)	140 ± 17	d (x)	997 ± 38	cd (xy)	92 ± 6	a (x)
24 h	CS1	4584 ± 198	a (w)	2744 ± 6	a (w)	11358 ± 263	a (w)	165 ± 8	a (w)
	CS2	854 ± 69	cd (wx)	491 ± 98	c (y)	1394 ± 578	cd (y)	186 ± 81	a (w)
	CS3	603 ± 185	d (y)	177 ± 60	d (z)	1248 ± 559	cd (y)	142 ± 24	a (w)
	CS4	1185 ± 155	b (x)	740 ± 62	b (x)	3179 ± 456	b (x)	118 ± 8	a (w)
**Significance**									
Cutting style (CS)		***		***		***			NS
Storage time (ST)		***		***		***			NS
CS × ST		***		***		***			NS
**Storage Time**	**Cutting Style ^A^**	**Glucosinolate Concentration (μmol/kg DW) ^B,C,D,E^**							
		**Gluconasturtiin**		**4-Methoxy Glucobrassicin**		**Neoglucobrassicin**		**Total Glucosinolates**	
0 h	CS1	595 ± 17	b (w)	120 ± 7	c (w)	365 ± 30	c (w)	3505 ± 298	c (w)
	CS2	491 ± 87	b (wx)	81 ± 24	cd (wx)	280 ± 36	c (wx)	2891 ± 587	cd (wx)
	CS3	295 ± 8.0	b (x)	71 ± 16	cd (x)	222 ± 33	c (x)	1757 ± 67	d (x)
	CS4	441 ± 111	b (wx)	40 ± 2	d (x)	194 ± 2	c (x)	1906 ± 71	cd (x)
24 h	CS1	3052 ± 210	a (w)	1155 ± 40	a (w)	1817 ± 65	a (w)	20289 ± 451	a (w)
	CS2	601 ± 265	b (x)	205 ± 13	b (x)	233 ± 17	c (x)	3032 ± 950	cd (y)
	CS3	487 ± 69	b (x)	99 ± 40	cd (y)	359 ± 161	c (x)	2512 ± 848	cd (y)
	CS4	3096 ± 383	a (w)	90 ± 7.5	cd (y)	606 ± 58	b (x)	7829 ± 964	b (x)
**Significance**									
Cutting style (CS)		**		***		***		***	
Storage time (ST)		***		***		***		***	
CS × ST		*		***		***		***	

^A^ Cutting styles (Figure 1): floret (CS1), floret cut into two even pieces (CS2), floret into four even pieces (CS3), florets processed into chops (CS4). ^B^ Concentrations were expressed as mmol of desulfosinigrin equivalents per kg of broccoli dry weight (DW). ^C^ Data represents the mean of three repetitions ± standard error of the mean. ^D^ Different letters in the same column indicate statistical difference by the LSD test (*p* < 0.05). (a-d) when comparing all treatments and storage times, (w-z) when comparing all treatments at the same storage time (0 h or 24 h). ^E^ Asterisks indicate that main effects and interactions are significantly different by analyses of variance (ANOVA). NS—non significant, * *p* < 0.05, ** *p* < 0.01, *** *p* < 0.001.
